# Mitochondrial DNA depletion, mitochondrial mutations and high TFAM expression in hepatocellular carcinoma

**DOI:** 10.18632/oncotarget.21033

**Published:** 2017-09-16

**Authors:** Lihua Qiao, Guoqing Ru, Zhuochao Mao, Chenghui Wang, Zhipeng Nie, Qiang Li, Yiyi Huang-yang, Ling Zhu, Xiaoyang Liang, Jialing Yu, Pingping Jiang

**Affiliations:** ^1^ Division of Medical Genetics and Genomics, The Children's Hospital, Zhejiang University School of Medicine, Hangzhou, China; ^2^ Institute of Genetics, Zhejiang University and Department of Genetics, Zhejiang University School of Medicine, Hangzhou, Zhejiang, China; ^3^ Department of Pathology, The Zhejiang Provincial People's Hospital, Hangzhou, China; ^4^ Department of Surgical Oncology, First Affiliated Hospital, School of Medicine, Zhejiang University, Hangzhou, China; ^5^ School of Public Health, Zhejiang University, Hangzhou, China

**Keywords:** mitochondrion, hepatocellular carcinoma, somatic mutation, DNA copy number, TFAM

## Abstract

We investigated the role of mitochondrial genetic alterations in hepatocellular carcinoma by directly comparing the mitochondrial genomes of 86 matched pairs of HCC and non-tumor liver samples. Substitutions in 637 mtDNA sites were detected, comprising 89.80% transitions and 6.60% transversions. Forty-six somatic variants, including 15 novel mutations, were identified in 40.70% of tumor tissues. Of those, 21 were located in the non-coding region and 25 in the protein-coding region. Twenty-two somatic nonsynonymous changes were identified as putative pathogenic variants, including 4 truncating mutations produced by three frameshifts (*MT-ATP6* 8628 insC; *MT-ND5* 13475 T-del, and *MT-CYB* 14984 insA) and 1 nonsense mutation in *MT-CO3* 9253 G>A. Among the somatic variants, only m.13676 A>G (*MT-ND5*), found in only 1 tumor, was heteroplasmic. Both inherited and somatic variants were predominately located in the D-loop region and the *MT-ND5* gene. Tumor/non-tumor paired analysis showed that 69% of HCC samples contained significantly reduced mtDNA, compared with 49.0% of non-tumor counterparts. In 81.40% of HCC samples, mitochondrial transcription factor A (TFAM) was enriched in tumor cells but not in adjacent non-tumor cells. Neither mtDNA depletion nor TFAM overexpression correlated with the degree of cell differentiation, though TFAM expression correlated with tumor size.

## INTRODUCTION

Mitochondria are complex organelles involved in many essential cellular processes. Besides their role in energy balance through oxidative phosphorylation (OXPHOS), mitochondria are crucial regulators of metabolism and apoptosis [1–3]. Since the generalized acceptance of the Warburg effect as the mechanism underlying decreased OXPHOS and increased aerobic glycolytic energy production in cancer cells, a strong correlation has been found between mitochondrial dysfunction, represented by mitochondrial protein and DNA (mtDNA) mutations and changes mtDNA content, and human cancers [4, 5].

Hepatocellular carcinoma (HCC) is the most common histological type of primary liver cancer and the third leading cause of cancer-induced mortality worldwide [6]. Prior studies have been shown that impairment of the OXPHOS system and excessive reactive oxygen species (ROS) production are the most important factors in HCC carcinogenesis [7]. Normal OXPHOS is controlled by both mtDNA and the nuclear genome. The mtDNA encodes 13 polypeptides of the OXPHOS complexes mediating electron transfer and ATP synthesis, 2 ribosomal RNA genes, and 22 transfer RNA genes involved in the translation of these 13 subunits [8]. Recently, several tumor-specific mtDNA somatic mutations were found in various cancers and proposed to contribute to tumorigenesis [9, 10]. These variants accumulated especially in the displacement loop (D-loop) region which controls replication and transcription of mtDNA [11–14]. However, a thorough characterization of tumoral mtDNA alterations has yet to be achieved, due to insufficient paired (tumor/normal) sample comparisons and incomplete analysis of the entire mitochondrial genome. Alterations in the D-loop have been suggested to correlate with mtDNA copy number in a tumor-specific manner, with decreased copy number found in bladder cancer, breast cancer, and HCC, and increased copy number detected in lung cancer and head and neck cancer, among others [15–18]. As the transcription and replication of mtDNA require proteins encoded by nuclear DNA, the mitochondrial transcription factor A (TFAM), a nuclear factor, was suggested to also contribute to tumorigenesis [19]. However, neither the pattern of TFAM expression in HCC nor its correlation to mtDNA content had been so far addressed.

To characterize changes in the mtDNA of HCC patients, we performed a comparative analysis of the entire mitochondrial genome of 86 matched HCC tumor and non-tumor liver tissues in order to characterize inherited and somatic variants, alterations in mtDNA copy numbers, and tumor-specific transcriptional signatures of genes involved in mtDNA maintenance.

## RESULTS

### Patient demographics

Eighty-six individuals diagnosed with HCC were enrolled in the present study. Of these, 69 (80.2%) were males and 17 (19.8%) were females (Supplementary Table 1), a gender distribution ratio consistent with a recent report [20]. The age at diagnosis ranged from 24 to 84 years, with a mean of 55.3 years. The distribution of cell differentiation (Edmondson and Steiner's grading, ED grade) [21] among patients was as follows: grade I, 10 (11.6%) patients; grade II, 61 (70.9%) patients; grade III, 14 (16.3%) patients, and grade IV, 1 (1.2%) patient. A larger proportion of tumors were located on the right (61.6%, 53/86) versus the left (29.1%, 25/86) liver lobes. No significant associations were found between ED grade and either gender or age.

### Characterization of inherited and somatic mtDNA variants

**Figure 1:**
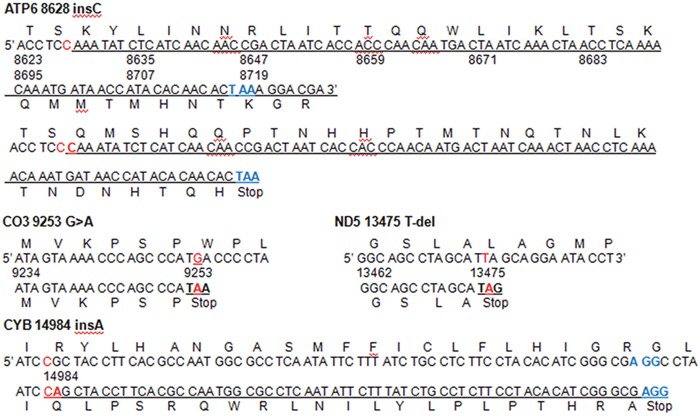
Four protein truncating mutations in mtDNA Three frameshifts (8628 insC, 13475 T-del, and 14984 insA) and one nonsense mutation (9253 G>A) introduce a stop-codon in DNA transcription. All changes are homoplasmic.

PCR amplification of fragments spanning the mitochondrial genomes followed by DNA sequence analysis were performed in 86 matched pairs of tumor and non-tumor liver tissues. Pairwise comparison of mtDNA sequences was used to identify inherited mtDNA variants shared by both sample types, and somatic variants occurring in tumor tissues only. A higher ratio of transition to transversion (ts/tv), consistent with previous reports [22, 23], was observed in the 637 mtDNA substitution sites detected. Among these, 572 (89.80%) were transitions and only 42 (6.60%) were transversions. In addition, 28 novel variants were found (Supplementary Tables 2-4). 36.04% (213/591) of the inherited variants were distributed among non-protein coding sequence regions, and included 22 tRNAs, 2rRNAs, the control region (D-loop), and other non-coding regions (Table 1). 378 (63.96%) variants were in protein-coding regions, including 258 synonymous and 120 nonsynonymous variants. 16 small (<10 bp) inherited indels were observed, all of which occurred in non-protein coding regions. Forty-six somatic substitutions, including 15 novel variants, were identified in 35 out of 86 tumor tissues and were absent in the matched non-tumor samples; of those, 21 substitutions were distributed in the D-loop (14), 12S rRNA (2), 16S rRNA (1), and tRNAs (4, in the *MT-TF, MT-TN*, *MT-TS2*, and *MT-TP* genes) (Table 2). A novel deletion (48bp) from position 294 to 341 was observed in two tumors, and a “CCCC” insertion in position 573 was detected in the D-loop region in 1 tumor. Twenty-five somatic variants were found within protein-coding regions, of which only 3 (12%) were synonymous changes. Meanwhile, 68.7% of inherited substitutions in the protein-coding region were synonymous. Twenty-two somatic variants were nonsynonymous, including 4 truncating mutations produced by three frameshift truncations (*MT-ATP6* 8628 insC; *MT-ND5 13475 T-del*, and *MT-CYB* 14984 insA) and one nonsense mutation in *MT-CO3* 9253 G>A (Figure 1). Furthermore, the 22 somatic nonsynonymous variants were evaluated by 6 bioinformatic programs, and predicted to be putative pathogenic mutations (Supplementary Table 5). Strikingly, both inherited and somatic variants were frequently accumulated in the D-loop region and the *MT-ND5* gene.

**Table 1:** Overview of inherited variants in 86 HCC pairs

Region/gene	Var no. (%)	Transitions	Transversions	del	ins	Non-syn	Syn	novel Var
Control region	128 (21.66%)	105	13	5	5			1
^*^other non-coding	9 (1.52%)	5	0	2	2			0
12S rRNA	21 (3.55%)	19	1	0	1			1
16S rRNA	24 (4.06%)	23	1	0	0			2
tRNAs	31 (5.25%)	28	2	0	1			4
MT-ND1	37 (6.26%)	35	2	0	0	12	25	1
MT-ND2	29 (4.91%)	26	3	0	0	11	18	0
MT-ND3	11 (1.86%)	11	0	0	0	4	7	0
MT-ND4	40 (6.77%)	38	2	0	0	9	31	0
MT-ND4L	6 (1.02%)	5	1	0	0	3	3	0
MT-ND5	62 (10.49%)	60	2	0	0	23	39	2
MT-ND6	13 (2.20%)	13	0	0	0	3	10	0
MT-CO1	38 (6.43%)	36	2	0	0	8	30	1
MT-CO2	24 (4.06%)	24	0	0	0	4	20	0
MT-CO3	27 (4.57%)	26	1	0	0	6	21	0
MT-CYB	57 (9.64%)	52	5	0	0	22	35	1
MT-ATP6	25 (4.23%)	25	0	0	0	11	14	0
MT-ATP8	9 (1.52%)	8	1	0	0	4	5	0
Total	591	539	36	7	9	120	258	13

^*^, regions interspersed between the coding gene and tRNAs.

**Table 2:** Somatic variants in HCC samples

Region/gene	Position	Replacement	Reported	AA change	Inter-species conservation^#^
Control region	71	G-A	Yes		
	72	T-C	Yes		
	74	T-G	Yes		
	94	G-A	Yes		
	157	T-C	No		
	294-341	Del	No		
	302	A-AC	Yes		
	353	C-CC	Yes		
	394	C-A	No		
	528	T-C	No		
	540	A-C	No		
	573	C-CCCCC	Yes		
	16368	T-C	Yes		
	16540	C-T	Yes		
*MT-TF*	617	G-A	No		
12S rRNA	988	G-A	Yes		
	1472	G-A	Yes		
16S rRNA	2623	A-G	Yes		
*MT-ND1*	3710	C-T	Yes	Ala-Val	16/17
	3877	T-C	No	Ala-Pro	17/17
*MT-ND2*	4963	G-A	No	Gly-Asp	17/17
	5112	G-A	Yes	Ala-Thr	5/17
*MT-TN*	5705	A-G	Yes		
*MT-CO1*	6582	G-A	No	Asp-Asn	17/17
	7347	G-A	Yes	Val-Ile	16/17
*MT-ATP8*	8369	C-G	Yes	Pro - Ser	17/17
*MT-ATP6*	^*^8628	C-CC	No	frameshift (stop)	12/17
*MT-CO3*	^*^9253	G-A	Yes	Trp-stop	17/17
	9670	A-G	Yes	Asn - Ser	13/17
*MT-ND3*	10365	G-A	Yes	Ala-Thr	13/17
*MT-ND4L*	10689	G-A	Yes	Gly-Ser	16/17
*MT-ND4*	11226	G-A	No	Gly-Asp	17/17
	11929	T-C	Yes	Syn	
*MT-TS2*	12209	G-A	Yes		
*MT-ND5*	12634	A-G	Yes	Ile-Val	14/17
	12711	A-G	Yes	Syn	17/17
	12954	T-C	Yes	Syn	17/17
	13063	G-A	Yes	Val-Ile	17/17
	13267	G-T	No	Gly-Trp	17/17
	^*^13475	T-Del	No	frameshift (stop)	17/17
	13603	A-G	No	Ser - Gly	17/17
	13676	A-G	Yes	Asn - Ser	15/17
	13718	G-A	Yes	Ser-Asn	17/17
*MT-CYB*	^*^14984	C-CA	No	frameshift (stop)	15/17
	15860	A-G	Yes	Ile - Val	12/17
*MT-TP*	16017	T-C	Yes		

^*^, four truncating mutations in the protein-coding region.

#, 17 species: *Bos taurus, Pan troglodytes, Cebus albifrons, Gorilla gorilla, Homo sapiens, Hylobates lar, Lemur catta, Macaca mulatta, Macaca sylvanus, Mus musculus, Nycticebus coucang, Pan paniscus, Pongo Pygmaeus, Tarsius bancanus, and Xenopus laevis*.

Conservation was calculated by comparing the human nucleotide variants with those of other 16 vertebrates, and defined as the percentage of species from the list of 17 different vertebrates that had the wild-type nucleotide at that position.

Although heteroplasmy is expected to play an important role in tumorigenesis [24], only 7 heteroplasmic mtDNA mutations were found in just 4 HCC patients. Five of these variants were observed in non-tumor tissues and 2 in tumor tissues (Supplementary Table 6). In addition, only 1 heteroplasmic somatic variant, m.13676 A>G (*MT-ND5*), was observed in 1 tumor specimen.

### Analysis of mtDNA content and haplogroup distribution

**Figure 2:**
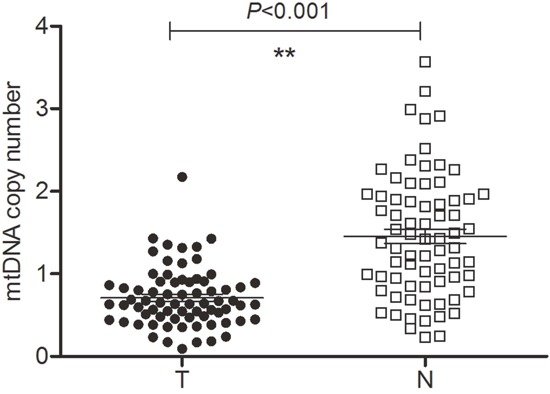
Relative mtDNA content in tumor tissues (T) and matched non-tumor tissues (N) Mean T mtDNA content was significantly lower (0.71 ± 0.042) than N (1.45 ± 0.088) (*P* < 0.001).

Since changes in mtDNA content have been frequently described in human tumors, we compared relative mtDNA contents in matched pairs of tumor and non-tumor tissues. As shown in Figure 2, the mean relative mtDNA copy number in tumor samples was markedly reduced, to only 49.0% of that in non-tumor samples (*P* < 0.001). Our analysis showed that 69% of tumor samples contained less mtDNA than their non-tumor counterparts.

The entire mtDNA sequences of the 86 HCC samples were assigned to the Asian mtDNA lineage and classified into 11 haplogroups distributed between the macro-haplogroups M (*n* = 42) and N (*n* = 44). Five sub-haplogroups (D, G, M7, M8 and M12) were derived from the macro-haplogroup M, and six from the macro-haplogroup N (A, B, N9, R9, R11 and H2) (Table 3). No remarkable bias was detected in the M/N haplogroup distribution in HCC samples (*P* = 0.553). Moreover, no significant association was found between mtDNA haplogroup and somatic variants (*P* = 0.755), or reduced mtDNA content (*P* = 0.879).

**Table 3:** Distribution of mtDNA haplogroup among HCC pairs/subjects

mtDNA haplogroup	M					N						*P*
	D	G	M7	M8	M12	A	N9	R9(F)	R11	B	H2	
HCC patients, n = 86	18	4	14	5	1	8	5	20	2	8	1	0.553
Subjects with somatic variants, n = 35	8	2	5	2	1	6	1	6	1	2	1	0.755
Tumor tissues with reduced mtDNA content, n = 50	10	2	8	3	1	5	2	13	1	5		0.879

### TFAM expression in paraffin tissues

**Figure 3:**
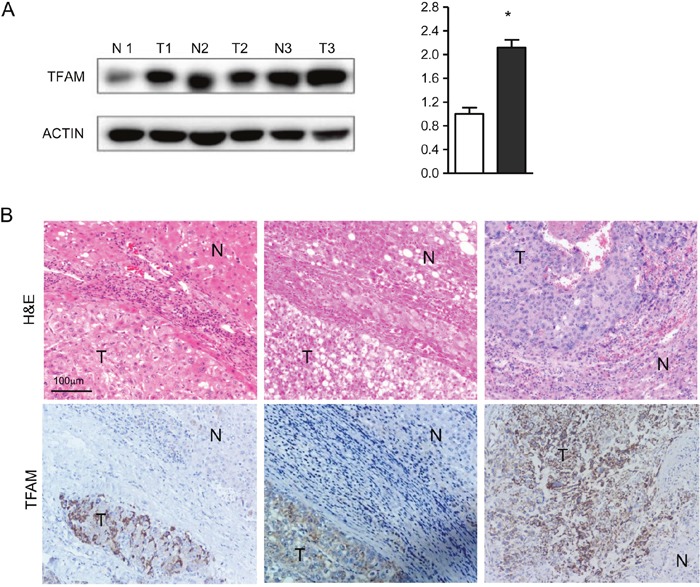
Overexpression of TFAM in HCC samples by **(A)** Western blotting analysis; and **(B)** IHC analysis in paraffin sections (bar: 100 mm) with H&E staining and antibody against TFAM. ‘T’ indicates tumor tissue or cells, while ‘N’ indicates non-tumor tissue or cells. Non-tumor tissues or adjacent non-tumor cells in the same section were used as negative controls.

Since somatic variants and depleted mtDNA were observed in HCC, we set out to ask if changes in mtDNA influenced the expression of genes, especially factors controlling mtDNA replication and transcription. Initial analysis of 3 tumor and non-tumor sample pairs revealed that TFAM protein levels were consistently higher in the tumor specimens (Figure 3A) (*P < 0.001*). To confirm this tendency, we further examined TFAM expression by IHC in a larger sample number (Figure 3B). Signal quantitation showed a marked enrichment in TFAM levels in tumor cells compared to adjacent normal liver cells in the same sections. As shown in Table 4, positive expression of TFAM was detected in 81.40% (70/86) of tumor samples, including 8/10 grade I, 48/61 grade II, 13/14 grade III, and 1/1 grade IV samples. The expression of TFAM was not correlated with cell differentiation grade, but was associated with tumor size (*P* = 0.038).

**Table 4:** TFAM expression in paraffin tissues and its correlation with some clinicopathologic factors

Variable	Total	TFAM expression		*P*
		Positive	Negative	
Patient number	86	70	16	
Age (year)				
<60	52	43	9	
≥60	34	27	7	0.702
Gender				
Male	69	56	13	
Female	17	14	3	0.91
ED Grade				
Grade I & II	71	56	15	
Grade III & IV	15	14	1	0.191
Tumor Size^#^				
≤5cm	51	38	13	
>5cm	35	32	3	**0.038**

#, Tumor Size (5 cm) list here according the Primary Tumor (T) of TNM Categories in WHO (http://www.who.int/iris/handle/10665/68618).

## DISCUSSION

The present study evaluated matched tumor and non-tumor samples to provide a comprehensive characterization of mtDNA alterations, further disclosing a significant elevation of TFAM expression in HCC.

It's universally accepted that cancer cells switch from OXPHOS to aerobic glycolysis (Warburg effect), which markedly increases their biosynthetic capacity and stimulates tumor growth. This has led to the controversial concept of mitochondrial dysfunction as a possible cause of tumorigenesis. Consequently, the molecular bases of mitochondrial dysfunction, especially the factors influencing mtDNA alterations, have been investigated in many tumor types. The D-loop region plays a crucial role in mtDNA replication and transcription and is a hotspot for mutations in human cancers because of its higher susceptibility to genetic changes, compared with the mtDNA coding region [8]. Consistent with precious reports [25, 26], both inherited and somatic substitutions were detected in the hypervariable D-loop regions in our 86 HCC pairs. Although common variants in the D310 (located between nt303 and nt315) mononucleotide repeat of mtDNA were detected in tumor and non-tumor liver tissues, 4977 deletions found in cancers elsewhere were not detected in this study [26–28]; in addition, the frequency of somatic mtDNA variants was different among tumor types. In this work, 40.67% of HCCs carried somatic variants, a frequency similar to that reported in HCC by Lee et al. [29] and Yin et al. [30], but lower than that of other type of cancers [31]. Strikingly, both the D-loop and the *MT-ND5* gene seemed to be hotspots for somatic variants in our HCC cases, as 30.43% (14/46) of variants were found in the D-loop region and 19.57% (9/46) in *MT-ND5*. Somatic D-loop variants mostly occurred in or near its conserved sequence blocks (CSBs), or in the heavy-strand promoter (HSP) to which TFAM binds. These included 302 insC and 353 insC in CSBs, and 540A>C at the TFAM biding site. We also found a 294-341 deletion in 1 tumor, comparable to a 298-348 deletion previously reported in HCC [30]. On the other hand, the *MT-ND5* gene has been reported to carry the majority of truncating mutations in colon or rectal adenocarcinoma [31], and was associated with mitochondrial respiratory chain deficiency [32]. However, the four truncating mutations in our study were observed in the *MT-ATP6, MT-CO3, MT-ND5*, and *MT-CYB* genes. Three of them were reported in HCC for the first time here, while *MT-CO3* 9253 G>A was previously detected in papillary thyroid cancer [18]. Furthermore, 22 somatic nonsynonymous changes in the protein-coding region, including the four truncating mutations referred above, were evaluated by bioinformatic programs to evaluate their potential pathogenicity.

MtDNA copy number varies across tumor types. Reduced mtDNA copy number has been reported in breast [15, 33] and kidney [34] cancers, as well as in HCC [26, 30]. In the present study, the mean mtDNA copy number in tumor tissues was significantly reduced (by half) compared with non-tumor tissues. Paired analysis of tumor/nontumor data showed that 69% of the HCC samples had lower mtDNA copy number than the corresponding non-tumor specimens. A similar frequency (60%) was reported by Yin et al. in HCC [26], and by Lee et al. in gastric cancers (54.8%) [35]. Interestingly, one of the HCC samples analyzed by us showed higher mtDNA content compared to its matched non-tumor counterpart, in which four heteroplasmic variants (16261C>C/T, 16311T>T/C, 4883 C>C/T, and 12092 C>C/A) were observed. We speculate that the increased tumoral mtDNA content might have resulted from a compensatory response elicited by the heteroplasmic variants in the early stage of tumorigenesis. Consistent with previous work [15, 36], no correlation between reduced mtDNA content and cell differentiation grade was observed in our study. Therefore, these findings suggest caution in the clinical use of mtDNA copy number alone as tumor biomarker.

The regulation of mtDNA copy number is complex and depends on several factors, including mtDNA mutations and genetic variations within mtDNA replication and transcription genes. A previous study reported high relative gene expression for TFAM and POLG in human astrocytomas [37]. Here, we found a significantly high expression of TFAM by western blotting, confirmed in 70 tumor tissues via IHC, with adjacent non-tumor cells used as negative control. TFAM signal was enriched in all tumor tissues with reduced mtDNA content. Nevertheless, some tumor cells with higher mtDNA content than the corresponding non-tumor cells were also positive for TFAM. This evidence suggests that overexpression of TFAM, the most abundant component of the mitochondrial nucleoid, may be a compensatory response to mtDNA instability caused by the somatic variants. ChIP-seq of TFAM-DNA revealed that TFAM coats the mitochondrial genome in a non-specific manner, increasing mtDNA integrity [38]. Lu et al. discovered that Lon protease knockdown transiently increased both TFAM levels and mtDNA copy number [39], implying that Lon knockdown may mimic abnormal degradation of TFAM in tumor cells. However, the expression of Lon in tumor tissues was inconsistent, being higher in some tumor tissues and lower in others, compared with their matched non-tumor counterparts (data not shown). Furthermore, no correlation could be established between TFAM expression and cell differentiation grade in this study. The expression of TFAM was shown to be correlated to the tumor size of HCC, one feature of TNM, though it was reported to be correlated to TNM stage in astrocytomas [19], colorectal cancer [40], and non-small cell lung cancer [41]. Further, *in vitro* experiments showed depletion of both TFAM and mtDNA in HeLa cells treated with ethidium bromide [42], and it was suggested that mtDNA depletion may also be a consequence of defects in mtDNA maintenance caused by mutations in nuclear genes [43]. In this regard, heterozygous *TFAM* mutations associated with mtDNA depletion were reported in some colorectal cancers [5]. Whereas this evidence suggests that depleted or enriched TFAM parallels changes in mtDNA and might be specific of certain cancer types, further investigation is needed to evaluate the influence of TFAM mutations in HCC development and progression.

Our data shows that both the D-loop region and the *MT-ND5* gene are hotspots for substitutions in HCC. Forty-six somatic variants were found in 35 tumor tissues, of which 15 were novel and 22 were nonsynonymous changes, including 4 truncating mutations, distributed in the protein-coding region of mtDNA. Most HCC tumor samples showed elevated expression of TFAM and depleted mtDNA content, compared to matched non-tumor specimens. Little heteroplasmy was observed in HCC samples in this study. The present results provide novel information that should be useful to advance our understanding of the role of mitochondria in hepatocellular carcinoma.

## MATERIALS AND METHODS

### Patients and tissue specimens

Eighty-six cases of HCC were enrolled between 2013 and 2014. 2 samples from each case, tumor tissue and non-tumor liver tissue, were proceeded pathological diagnosis, which performed according to the 2010 WHO classification. All the cases were diagnosed as HCC-classical and none of them was combined hepatocellular carcinoma-cholangiocarcinoma. The tumor and matched non-tumor liver tissues were resected at Zhejiang Provincial People's Hospital after informed consent was obtained. Samples were fixed in 10% buffered formalin, followed by paraffin embedment. Tissue sections were independently evaluated by two experienced pathologists according to the Edmondson and Steiner's grading (ED grade) [21]. This study was approved by the Ethics Committee of the Zhejiang Provincial People's hospital (protocol KY2015261).

### Analysis of the mitochondrial genome

Total DNA was isolated from 86 pairs of tumors (T) and matched non-tumor (N) liver tissues using a Tissue gDNA kit (Biomiga) according to the manufacturer's protocol. The entire mitochondrial genome of the 86 pairs was PCR amplified in 24 overlapping fragments using sets of oligonucleotide primers as described previously [44]. Each fragment was purified and subsequently analyzed by Sanger sequencing. Sequencing results were compared with the updated Cambridge Reference Sequence (GenBank accession number: NC_012920) [45], along with the Human Mitochondrial Genome Database (mtDB) and the Mitomap database [http://www.mitomap.org/MITOMAP]. Twenty-two somatic nonsynonymous variants in the protein-coding region, with a frequency <0.5% in the Mitomap database, were further evaluated by bioinformatic programs including PolyPhen-2 (http://genetics.bwh.harvard.edu/pph2/), SIFT (http://sift.jcvi.org/), MutationAssessor (http://mutationassessor.org/), Provean (http://provean.jcvi.org/index.php), PANTHER (http://fathmm.biocompute.org.uk/), MToolBox (https://github.com/mitoNGS/MToolBox), and TransFIC (http://bg.upf.edu/transfic/home). Variants that were predicted as deleterious by more than half of the 6 programs listed above were considered as putative pathogenic mutations associated with HCC (Supplementary Table 5). Records of the 22 somatic nonsynonymous variants were sought in the COSMIC database (http://cancer.sanger.ac.uk/cosmic). The entire mtDNA sequences of the 86 patients were assigned to the Asian mitochondrial haplogroups by using the nomenclature of mitochondrial haplogroups [46].

### Measurement of mtDNA copy number

The copy number of mtDNA was determined by comparing the ratio of mtDNA to nDNA (β-actin) by real-time quantitative PCR as described previously [47]. The D-loop fragment was used as reference to determine the amount of mtDNA. The relative ratio was analyzed on a 7900HT system (Applied Biosystems) using FastStart Universal SYBR Green Master Mix (Roche Diagnostics GmbH). Experiments were repeated in triplicate. Following mtDNA sequencing, 72 pairs of copy numbers from tumor/non-tumor DNA samples were plotted with GraphPad Prism software as shown in Figure 2.

### Immunohistochemistry

Paraffin-embedded sections were stained with hematoxylin and eosin (H&E), and immunohistochemistry (IHC) was performed using an anti-TFAM antibody (Abcam). The degree of immunostaining was evaluated semi-quantitatively by combining two staining scores (intensity and surface area) as described elsewhere [48]. Staining intensity was scored as: 0 (no staining), 1 (weak staining, light yellow), 2 (moderate staining, yellow brown), and 3 (strong staining, brown). The proportion of stained tumor cells was graded from 0 to 4 according to the distribution area of positive cells as: 0, no staining; 1, 1–5% positive cells; 2, 6–25% positive cells; 3, 26–50% positive cells; and 4, >50% positive cells. Equivocal reaction was regarded as negative.

### Statistical analysis

All statistical analyses and graphical data were generated using GraphPad Prism software (v5.04, www.graphpad.com) or Microsoft-Excel, with data presented as Mean ± SD. The *P* value of mitochondrial haplotype was calculated using IBM SPSS Statistics 20. Statistical significance was established at *P* < 0.05 (^*^) and *P* < 0.01 (^**^).

## SUPPLEMENTARY MATERIALS FIGURES AND TABLES









## Abbreviations

HCC: hepatocellular carcinoma
mtDNA: mitochondrial DNA
OXPHOS: oxidative phosphorylation
ROS: reactive oxygen species
D-loop: displacement loop
TFAM: mitochondrial transcription factor A
POLG: DNA polymerase γ
IHC: immunohistochemistry
SD: standard deviation
