# Supplementary material for: Mitochondrial DNA depletion, mitochondrial mutations and high TFAM expression in hepatocellular carcinoma
Source: Oncotarget. 2017 Sep 16;8(48):84373–83. doi: 10.18632/oncotarget.21033 (PMC5663603; doi:10.18632/oncotarget.21033)
Supplement: Supplementary file 1 [file oncotarget-08-84373-s001.pdf]

# Mitochondrial DNA depletion, mitochondrial mutations and high TFAM expression in hepatocellular carcinoma

## SUPPLEMENTARY MATERIALS

**Supplementary Table 1: Information of 86 HCC subjects and TFAM expression**

See Supplementary File 1

**Supplementary Table 2: Summary information of substitutions in 637 mtDNA sites among 86 HCC pairs**

See Supplementary File 2

**Supplementary Table 3: Overview of mtDNA variants in 86 HCC pairs**

| Region/gene | Var no.(%) | Transitions | Transversions | del | ins | Non-syn | Syn | novel Var |
|---|---|---|---|---|---|---|---|---|
| Control region | 142 (22.29%) | 112 | 16 | 6 | 8 | | | 6 |
| other non-coding | 9 (1.41%) | 5 | 0 | 2 | 2 | | | 0 |
| 12S rRNA | 23 (3.61%) | 21 | 1 | 0 | 1 | | | 1 |
| 16S rRNA | 25 (3.92%) | 24 | 1 | 0 | 0 | | | 2 |
| tRNAs | 35 (5.49%) | 32 | 2 | 0 | 1 | | | 5 |
| MT-ND1 | 39 (6.12%) | 37 | 2 | 0 | 0 | 14 | 25 | 2 |
| MT-ND2 | 31 (4.87%) | 28 | 3 | 0 | 0 | 13 | 18 | 1 |
| MT-ND3 | 12 (1.88%) | 12 | 0 | 0 | 0 | 5 | 7 | 0 |
| MT-ND4 | 42 (6.59%) | 40 | 2 | 0 | 0 | 10 | 32 | 1 |
| MT-ND4L | 7 (1.10%) | 6 | 1 | 0 | 0 | 4 | 3 | 0 |
| MT-ND5 | 71 (11.15%) | 66 | 4 | 1 | 0 | 30 | 41 | 5 |
| MT-ND6 | 13 (2.04%) | 13 | 0 | 0 | 0 | 3 | 10 | 0 |
| MT-CO1 | 40 (6.28%) | 38 | 2 | 0 | 0 | 10 | 30 | 2 |
| MT-CO2 | 24 (3.77%) | 24 | 0 | 0 | 0 | 4 | 20 | 0 |
| MT-CO3 | 29 (4.55%) | 28 | 1 | 0 | 0 | 8 | 21 | 0 |
| MT-CYB | 59 (9.26%) | 53 | 5 | 0 | 1 | 24 | 35 | 2 |
| MT-ATP6 | 26 (4.08%) | 25 | 0 | 0 | 1 | 12 | 14 | 1 |
| MT-ATP8 | 10 (1.57%) | 8 | 2 | 0 | 0 | 5 | 5 | 0 |
| Total | 637 | 572 | 42 | 9 | 14 | 142 | 261 | 28 |

Var: variants; Syn: synonymous.

**Supplementary Table 4: Novel variants in HCC pairs**

| Region/ Gene | Position | Replace ment | Codon | Amino change | 17 species conservation | haplotype | T/N | Hom/Het |
|---|---|---|---|---|---|---|---|---|
| D-loop | 157 | T-C | | | | A4 | T | Hom |
| | 294-341 | 48bp-del | | | | F1a1c | T | Hom |
| | 394 | C-A | | | | F1a1c | T | Hom |
| | 528 | T-C | | | | F1a1d | T | Hom |
| | 540 | A-C | | | | F1a1d | T | Hom |
| | 568 | C-CC | | | | G2a2 | TN | Hom |
| MT-TF | 617 | G-A | | | | F1a1c | T | Hom |
| 12S rRNA | 955 | A-AC | | | | M7c3b | N | Hom |
| 16S rRNA | 2864 | T-C | 1194 | | | F1a1c | TN | Hom |
| | 3222 | C-A | 1551 | | | M7c3 | TN | Hom |
| MT-ND1 | 3877 | T-C | 191 | Ala-Pro | 17/17 | F1a1c | T | Hom |
| | 4153 | G-A | 283 | Asp-Asn | 17/17 | A4 | TN | Hom |
| MT-TM | 4468 | T-A | 72 | | | M7b1 | TN | Hom |
| MT-ND2 | 4963 | G-A | 165 | Gly-Asp | 17/17 | F1a1c | T | Hom |
| MT-CO1 | 5942 | A-G | 13 | Syn | 17/17 | F1a | TN | Hom |
| | 6582 | G-A | 227 | Asp-Asn | 17/17 | F1a1c | T | Hom |
| MT-TS1 | 7509 | T-TT | 6 | | | D5a2a | TN | Hom |
| MT-ATP6 | 8628 | C-CC | 34 | Stop-gain | 12/17 | D4i | T | Hom |
| MT-TG | 10041 | A-C | 55 | | | F1a1d/A17 | TN | Hom |
| MT-ND4 | 11226 | G-A | 156 | Gly-Asp | 17/17 | F1a1c | T | Hom |
| MT-TS2 | 12214 | C-T | | | | M7 | TN | Hom |
| MT-ND5 | 13267 | G-T | 311 | Gly-Trp | 17/17 | F1a1c | T | Hom |
| | 13475 | T-Del | 380 | Leu-Stop | 17/17 | A4/M7c3 | T/T | Hom |
| | 13603 | A-G | 423 | Ser-Gly | 17/17 | F1a1a/F1a1c/N9a | T/TN/T | Hom |
| | 13718 | G-A | 461 | Ser-Asn | 17/17 | F1a1 | T | Hom |
| | 13754 | C-A | 473 | Ser-Tyr | 17/17 | F8a2 | TN | Hom |
| MT-CYB | 14765 | A-T | 7 | Thr-Ser | 8/17 | A4/N9a1/D4i | N/N/T | Hom |
| | 14984 | C-CA | 80 | Stop-gain | 15/17 | F2a | T | Hom |

T/N: T for tumor tissue, N for non-tumor tissue.
Hom/Het:Hom for homoplasmy, Het for heteroplasmy.

**Supplementary Table 5: Putative pathogenic variants identified in mtDNA protein coding region in HCC samples**

See Supplementary File 3

**Supplementary Table 6: Heteroplasmic variants in HCC pairs**

| Gene | Position | Replacement | aa change | Tumor tissue | | Non-tumor tissue | |
|---|---|---|---|---|---|---|---|
| | | | | Het (No.) | Hom (No.) | Het (No.) | Hom (No.) |
| D-loop | 16213 | G-A | | A/G (1) | *A (3) | 0 | A (2) |
| D-loop | 16261 | C-T | | 0 | T (6) | C/T | T (6) |
| | 16311 | T-C | | 0 | C (5) | T/C | C (5) |
| MT-RNR2 | 1736 | A-G | | 0 | G (8) | A/G | G (6) |
| MT-ND2 | 4883 | C-T | Thr-Ala | 0 | T (16) | C/T | T (16) |
| MT-ND5 | 12092 | C-A | Leu-Ile | 0 | 0 | C/A | 0 |
| MT-ND5 | 13676 | A-G | Asn - Ser | A/G (1) | 0 | 0 | 0 |

*A (3): adenine was occurred in 3 tumor tissues.
